# Endocrine Crosstalk Between Skeletal Muscle and the Brain

**DOI:** 10.3389/fneur.2018.00698

**Published:** 2018-08-24

**Authors:** Julien Delezie, Christoph Handschin

**Affiliations:** Biozentrum, University of Basel, Basel, Switzerland

**Keywords:** physical exercise, myokines, metabolites, BDNF, PGC-1α, hippocampus, memory, angiogenesis

## Abstract

Skeletal muscle is an essential regulator of energy homeostasis and a potent coordinator of exercise-induced adaptations in other organs including the liver, fat or the brain. Skeletal muscle-initiated crosstalk with other tissues is accomplished though the secretion of myokines, protein hormones which can exert autocrine, paracrine and long-distance endocrine effects. In addition, the enhanced release or uptake of metabolites from and into contracting muscle cells, respectively, likewise can act as a powerful mediator of tissue interactions, in particular in regard to the central nervous system. The present review will discuss the current stage of knowledge regarding how exercise and the muscle secretome improve a broad range of brain functions related to vascularization, neuroplasticity, memory, sleep and mood. Even though the molecular and cellular mechanisms underlying the communication between muscle and brain is still poorly understood, physical activity represents one of the most effective strategies to reduce the prevalence and incidence of depression, cognitive, metabolic or degenerative neuronal disorders, and thus warrants further study.

## Introduction

### Exercise is powerful medicine

Physicians increasingly encourage patients to exercise through medical prescriptions of specific physical activity programs based on the strong epidemiological link between a sedentary life style and various diseases (1–4). The deleterious effects of physical inactivity often are further exacerbated by excessive calorie intake resulting in obesity (5, 6). Nevertheless, a sedentary life-style is a strong and independent risk factor for a range of medical conditions such as cardiovascular pathologies, diabetes, certain types of cancer, depression, neurological disorders or stroke, for which the prevalence can be significantly reduced by being active and fit (7–9). However even though the epidemiological evidence is strong, our knowledge of the molecular and cellular mechanisms by which exercise promotes health is still rudimentary.

### The science of exercise

In terms of weight, skeletal muscle is the most abundant organ of the human body. Its function encompasses the maintenance of postural support, the generation of force and power during voluntary movements, breathing and thermoregulation. In addition, skeletal muscle accounts for a large portion of oxidative metabolism and of insulin-stimulated glucose uptake. Moreover, as a major storage site for glucose, lipids and amino acids, muscle is an essential coordinator of whole-body energy metabolism. For example, muscle protein metabolism rapidly adapts in response to physical exercise, dietary protein or anabolic hormones such as insulin-like growth factor 1 or testosterone (10, 11). The plasticity of skeletal muscle is further illustrated by the myriad of effects that either endurance- or resistance-based training induce at the cellular and molecular levels (12, 13). Recent observations in rodents and humans have demonstrated the ability of contracting myofibers to produce and release cytokines and other peptides, so-called myokines, which play a crucial role in the skeletal muscle crosstalk with other tissues and, together with muscle-controlled synthesis and degradation of metabolites, represent an essential mechanism to control whole-body homeostasis (14).

## Exercise response and the involvement of muscle PGC-1α

Exercise rapidly triggers substantial changes at the organismal level depending on various factors, including type, duration and intensity (13). To optimize contractions, the activity of the cardiovascular, respiratory, metabolic and neuroendocrine systems is modulated. At least in part, these adaptations are initiated from muscle tissue, for example by the paracrine release of nitric oxide (NO), ATP or reactive oxygen species (ROS) by myofibers (15). Other changes are mediated by hormonal signaling, e.g., by glucagon, catecholamines or growth hormones. For example, hepatic gluconeogenesis and lipolysis in adipose tissue are stimulated to ensure adequate energy supply to contracting myofibers (16).

In response to exercise, glycolysis products (i.e., lactate, pyruvate), lipolysis products (i.e., glycerol), amino acids (i.e., alanine and glutamine) and ketone bodies (in particular β-hydroxybutyrate; BOHB) are found at higher levels in the circulation (17, 18). Some of these metabolites, for example lactate and BOHB, are energy substrates for the central nervous system in the context of limited glucose (19, 20). BOHB and lactate transport is mediated by monocarboxylate transporters (MCTs) at the blood-brain barrier (21). Interestingly, exercise has been associated with changes in the expression of such transporters (MCT1, 2 and 4) in the rat cortex and hippocampus (22). Notably, lactate and BOHB are not only metabolic fuels for neuronal cells, but also bind the hydroxycarboxylic acid receptors (HCARs) and behave as signaling molecules at the central level (23). For instance, lactate influences neuronal activity, calcium signaling, axonal myelination, angiogenesis and memory formation (24, 25). The most abundant free amino acid in the human body, glutamine, essential as metabolic fuel and a protein building block, is a potent regulator of multiple signaling pathways related to inflammation, cell integrity and metabolism (26, 27). Interestingly, an elevation of glutamine in the rat hippocampus, striatum, and cerebellum following endurance exercise has recently been demonstrated (28). However, since the brain is autonomous for the production of glutamine and the blood-brain barrier rather impermeable to this amino acid, a redistribution from skeletal muscle in this context is rather unlikely (29). Lastly, it is important to highlight the metabolic coupling between neuronal and glial cells. In particular, prolonged exercise diminishes brain glycogen essentially stored in astrocytes providing lactate to adjacent neurons (30, 31). This lactate shuttle from astrocytes to neurons has been implicated in long-term memory formation (32), and has been shown to influence endurance capacity (33).

Exercise-induced cellular stress (e.g., ATP and O_2_ depletion, Ca^2+^ elevation, changes in the NADH/NAD^+^ ratio and mechanical stress) leads to the activation of multiple signaling pathways in skeletal muscle (16). Therefore, exercise is not only a potent modifier of the metabolome as previously discussed, but also affects the muscle epigenome, transcriptome and proteome (34–39). A marked acute exercise-induced hypomethylation of the promoters of the peroxisome-proliferator activated receptor γ coactivator 1α (PGC-1α) and the peroxisome-proliferator activated receptor δ (PPARβ/δ) in human muscle (34) parallels an upregulation of PGC-1α mRNA and protein stability, in part via PPARδ-dependent mechanisms (40–43). This PGC-1α-PPARδ axis plays a pivotal role in the regulation of muscle mitochondrial metabolism in the initial phase of exercise, and mediates the increase in mitochondrial biogenesis essential for endurance training adaptation. Importantly, PGC-1α acts as a central regulator of mitochondrial dynamics by recruiting and co-regulating multiple transcription factors including the nuclear respiratory factors NRF-1 and NRF-2, PPARα and PPARδ, and the estrogen-related receptor α (ERRα) (44–46). The PGC-1α-ERRα association is essential to coordinate lactate homeostasis during muscle contraction (47) and mediates exercise-induced angiogenesis through the induction of VEGF (48, 49). The active coordination of muscle angiogenesis by PGC-1α is further illustrated by its ability to induce the release of the secreted phosphoprotein 1 (SPP1), which recruits macrophages to help orchestrate multicellular angiogenesis (50). PGC-1α can also promote remodeling at the neuromuscular junction, which may be mediated by the secretion of neurturin (51, 52). Overall, PGC-1α integrates various cell signals during exercise.

The exercise-induced perturbations in the muscle milieu also lead to the activation of upstream kinases e.g., AMP-activated protein kinase (AMPK) and deacetylases e.g., SIRT1, that functionally interact with PGC-1α (53, 54). AMPK is a conserved energy sensor that is present in all mammalian cells, specifically activated in conditions that elevate the AMP:ATP ratio, with exercise being one of the most powerful activators of AMPK especially in human muscles (55). Acute exercise induces AMPK phosphorylation and enzymatic activity in an intensity-dependent manner, which in turn stimulates ATP production by promoting glucose transport and fatty-acid oxidation (16). Noteworthy, peripheral administration of the AMPK agonist AICAR can enhance running endurance capacity in sedentary mice. Likewise, a PPARδ agonist (GW501516) induces a gene program in skeletal muscle related to oxidative metabolism similar to endurance exercise (56). Whether these and related compounds are real “exercise mimetics” remains debatable (57). Intriguingly, treatment of wild-type mice with AICAR, and to a lesser extent with GW501516, enhances spatial memory and elevates neurogenesis in the hippocampal dentate gyrus area, again similar to exercise (58). Surprisingly, the effect of AICAR on memory function and hippocampal plasticity is dependent on the expression of the AMPK α2-subunit in muscle cells, providing evidence for a muscle-mediated mechanism (59).

## The wheel of health: neurotrophic and growth factors

In humans, the neurobiological effects of exercise are numerous e.g., reduction of anxiety and depression, improvement of social skills and self-esteem as well as mood and cognitive abilities (60, 61), in concert with significant structural changes at the central level (62–64). Voluntary exercise in a running wheel promotes neurogenesis and long-term potentiation in the adult rodent dentate gyrus (65, 66). Transcriptomic studies have described changes in gene categories related to chromatin remodeling, neuronal signaling and plasticity, protein synthesis and trafficking, and the inflammatory/immune response in rodent hippocampus after running [reviewed in (67)].

Importantly, one hallmark of physical activity is the central and peripheral induction of the brain-derived neurotrophic factor (BDNF). BDNF is a secretory growth factor belonging to the family of neurotrophins, which supports neural survival, growth, and synaptic plasticity. BDNF can signal via both TrkB and p75^NTR^ receptors (68–71) and is induced in the hippocampus of exercised rats (72). Inhibition of the action of BDNF in the central nervous system results in a reduction of the recruitment of the cAMP response-element-binding protein (CREB) to target sites on genes that mediate the exercise-induced enhancement in learning and memory (73). Moreover, there is evidence that physical activity in humans also elevates the level of circulating BDNF (74, 75). Similarly, BDNF brain and blood levels also correlate in other species i.e., pigs, rats, and humans (76). Notably, besides the brain as a potential source, a substantial amount of circulating BDNF originates from megakaryocytes, precursors of blood platelets; hence, the variations in BDNF in exercise studies could be caused by the different activation level of human platelets (77). The contribution of platelets to the exercise effects, in particular on the brain, remains unexplored (78), even though BDNF could be transported in platelet-derived vesicles to the brain (79). Unlike human megakaryocytes, this cell population is not involved in BDNF synthesis in the mouse (77). In contrast, BDNF is produced and secreted by human and rodent skeletal muscles and is regulated by exercise (80, 81). Muscle BDNF seems mostly involved in autocrine and paracrine signaling to promote muscle fiber fat oxidation and potentially muscle development (81–83), as well as in the retrograde signaling on motor neurons located in the spinal cord (84). Whether muscle-derived BDNF also signals to the brain, e.g., via TrkB signaling at the blood-brain barrier (85), remains to be investigated.

The blood-brain barrier consists of endothelial cells, vascular smooth cells, pericytes, basement membranes and astrocytic feet along cerebral microvessels, regulating the exchange between blood and neural tissue. This neurovascular interface acts as a barrier, limiting or facilitating the entry of specific nutrients, metabolites and hormones. For example, the hormone insulin-like growth factor-1 (IGF-1), mainly produced by the liver, can cross the blood-brain barrier and act as a mediator of the exercise-induced changes in hippocampal neurogenesis and BDNF expression (86, 87). Importantly, the formation and maintenance of dendritic spines in hippocampal neurons in basal conditions depend on the BDNF-dependent induction of PGC-1α after exercise (88, 89). In neuronal tissue, PGC-1α is a potent suppressor of ROS and thus exerts neuroprotective effects (90). Interestingly, long-term voluntary running exercise can slow down the progression of some neurodegenerative or age-related central deteriorations in mice (91–93). This is consistent with data emerging from human studies, in which aerobic exercise moderately prevents or delays the onset of aging- and neurodegeneration-associated memory loss (94–96). Noteworthy, neurogenesis in rodents occurs within an angiogenic niche (97) and exercise-induced vascularization is mediated in part by circulating vascular endothelial growth factor [VEGF; (98)]. Moreover, peripheral blockade of VEGF abolishes running-induced neurogenesis (99). Importantly, other neurotrophic factors are regulated by physical exercise in rodents. For instance, the expression of the glial cell line-derived neurotrophic factor or neurotrophin-3 is affected by physical exercise in skeletal muscle, the spinal cord, or the brain (100, 101, 102, 103). These results thus suggest that molecules other than BDNF could also mediate the neuroprotective effects of physical exercise at the central level [discussed in (104)].

Altogether, a combination of blood-born factors and centrally-expressed genes is required to promote angiogenesis and neurogenesis at the central level. Future studies are needed to characterize how IGF-1, VEGF and, putatively, BDNF signaling is integrated by neurons, and how exercise impacts the endothelial and glial cell populations at the blood-brain interface.

## From skeletal muscle to the brain: involvement of myokines and metabolites

Since the discovery of interleukin-6 (IL-6) as a cytokine produced by and released from skeletal muscle (105), various other exercise-induced factors have been identified by biochemical (106, 107, 108), proteomic (109, 110), or transcriptomic approaches (106, 111). These factors, called myokines, have autocrine, paracrine and/or endocrine effects [for review, see (14, 112)]. Importantly, the muscle secretome is not limited to protein hormones, as muscle cells also release or remove circulating metabolites. For instance, forced overexpression of PGC-1α in myocytes triggers the production and release of β-aminoisobutyric acid (BAIBA), which modulates both liver and fat metabolism (113). In this review, we focus on the recently described mediators of the muscle-to-brain communication (Table 1).

**Table 1:** Overview of muscle-derived and exercise-induced endocrine signals involved in the periphery-brain crosstalk.

**Protein or metabolite**	**Tissue**	**Experimental models**	**Detected in blood**	**Transport/receptor**	**Central effects**	**Main references**
**IDENTIFIED AND PUTATIVE MUSCLE-DERIVED FACTORS TRIGGERED BY EXERCISE**						
Cathepsin B	Skeletal muscle	CTSB KO mice Rat myotubes + AICAR treatment Peripheral CTSB injection	Human Rhesus Monkey Mouse	BBB crossing/ ?	↑ Hippocampal neurogenesis + spatial memory ↑ Hippocampal BDNF + DCX	(114)
Irisin	Brain Skeletal muscle	PGC-1α KO mice Adenovirus-mediated overexpression of PGC-1α (neurons) and FNDC5 (liver)	Human Mouse	BBB crossing/ ?	↑ Hippocampal BDNF	(89)
L-Lactate	Skeletal muscle	HCAR1 KO mice Exogenous L-lactate injection	Human Mouse	MCTs/HCAR1 at the BBB	↑ Vascularization ↑ VEGFA expression and vascularization	(115)
?	Skeletal muscle	Peripheral AICAR injection Deletion of the AMPK α2-subunit in mouse muscle	?	?	↑ Hippocampal neurogenesis + spatial memory	(58, 59)
**Protein or metabolite**	**Tissue/stimulus**	**Experimental models**	**Detected in blood**	**Transport/receptor**	**Central effects**	**Main references**
**ADDITIONAL FACTORS (NOT NECESSARILY INDUCED BY PHYSICAL ACTIVITY OR DERIVED FROM SKELETAL MUSCLE)**						
β-hydroxybutyrate	Liver (fasting + PA)	Exercise mouse Central β-hydroxybutyrate injection shRNA-mediated KD of HDAC3	Human Mouse	MCTs and HCARs at the BBB	↓ HDAC2 and HDAC3 recruitment at *Bdnf* promoters ↑ Hippocampal BDNF ↑ Glutamate release	(116)
Fibroblast growth factor 21	Liver (PA) Adipose Skeletal muscle	Peripheral and central FGF21 injection Overexpression of FGF21 in mouse liver Deletion of β-Klotho in mouse brain	Human Mouse	BBB crossing/βKlotho, FGFR1	Regulation of metabolism, energy expenditure, and circadian behavior	(117–119)
Insulin-like growth factor-1	Liver (PA)	Exercise rat Peripheral IGF-1 injection Anti-IGF-1 antibody + IGF-1 receptor antagonist	Human Rat Mouse	BBB crossing/IGF-1 receptor at the BBB	↑ Hippocampal neurogenesis and BDNF expression	(86, 87, 120)
Kynurenin (KYN) Kynurenic acid (KYNA)	Liver Skeletal muscle (PA)	Exercise mouse Overexpression/deletion of PGC-1α in mouse muscle Peripheral KYN injection	Human Mouse	KYN can cross the BBB but not KYNA/ ?	↓ KYN accumulation decreases stress-induced neurobiological mechanisms of depression	(121, 122)
?	Skeletal muscle	Mouse global, brain- or muscle-specific deletion/overexpression of BMAL1	?	?	Regulation of NREM sleep time and sleep recovery following sleep deprivation	(123)

*BBB, blood-brain barrier; PA, physical activity*.

### Irisin

In 2002, two studies reported the discovery of a cDNA encoding a novel protein, PeP/Frcp2, now named FNDC5 (124, 125). The secreted form of FNDC5, irisin, is a PGC-1α-dependent myokine induced by exercise (126). Noteworthy, irisin circulates at levels close to essential metabolic hormones (e.g., insulin, leptin) in humans (127), and acts on subcutaneous fat, promoting the beigeing and thermogenic program of white adipocytes, thereby increasing energy expenditure (126, 128). Moreover, irisin has the potential to modulate muscle and liver cell metabolism *in vitro* [reviewed in (129)]. Interestingly, FNDC5 has been detected in different areas of the brain (124, 125) and has been associated with neural differentiation (130). Additional evidence implies that FNDC5/irisin is also a part of the transcriptional response to exercise in the mouse hippocampus along with PGC-1α and BDNF (89). Mechanistically, the study demonstrated that the PGC-1α-dependent induction of FNDC5 in both primary cortical and hippocampal neurons involves the transcription factor ERRα. Moreover, FNDC5 is required to induce BDNF in a cell-autonomous manner, and recombinant BDNF treatment decreased FNDC5 expression as a part of a negative feedback loop. Most strikingly, the elevation of circulating levels of irisin by adenoviral overexpression of FNDC5 in the liver increased BDNF expression in the mouse hippocampus, but not in the forebrain.

Collectively, these data reveal a cellular mechanism by which endurance exercise promotes hippocampal gene expression such as BDNF and PGC-1α related to neuroprotection and memory. While it is unclear whether peripheral or central irisin mediate these effects in the brain, the adenoviral overexpression raises the possibility that circulating irisin could cross the blood-brain barrier, or act on receptors expressed by endothelial cells, to further amplify the effect of exercise on the central nervous system. In that regard, the deletion of FNDC5 in brain or skeletal muscle cells, together with the identification of its receptor, may delineate the exact contribution of systemic vs. local/central irisin. This seems especially important as modulation of central vs. peripheral irisin levels differently affects blood pressure in rodents (131).

### Cathepsin B

The cysteine protease cathepsin B (CTSB) is ubiquitously expressed throughout the body, including human muscle cells during exercise (109, 132). In the search of muscle-produced factors using a mass spectrometry-based approach, Moon et al. (114) exposed rat myotubes to the AMPK agonist AICAR and identified CTSB as a putative myokine. The authors further demonstrated that short-term AICAR treatment was effective in inducing *Ctsb* mRNA in myotubes. Moreover, 30 days of wheel-running activity upregulated *Ctsb* mRNA in the hippocampus and the gastrocnemius muscle (but not in other organs e.g., liver or adipose tissue), as well as plasma levels of CTSB in mice. Likewise, 4 months of treadmill exercise led to a significant increase of plasma CTSB concentrations in rhesus monkeys and humans. Furthermore, Moon et al. (114) showed that CTSB has the property to cross the blood-brain barrier and the application of exogenous CTSB to hippocampal progenitor cells induced both BDNF and doublecortin (DCX) transcripts. Strikingly, physical activity did not promote hippocampal neurogenesis or improved spatial memory in whole-body CTSB KO mice, suggesting that central and/or muscle-derived CTSB could mediate the beneficial effects of physical activity on memory function in rodents. Whether a correlation between human plasma CTSB levels and fitness or memory scores exists, as suggested by the authors, remains to be confirmed. Likewise, it is not clear if a therapeutic elevation of blood CTSB levels would be sufficient to enhance hippocampal-dependent memory function, especially in the aging context. Besides, the physiological effects of chronic exogenous CTSB administration have to be addressed, in particular in light of its involvement in inflammation, cell apoptosis, tumor development and progression (133, 134). Lastly, the signaling pathway by which peripheral and/or central CTSB promotes BDNF elevation and neurogenesis is still unknown.

### L-lactate

A recent study has uncovered a cellular mechanism by which blood-born lactate from contracting muscles signals to the brain to promote angiogenesis. Morland et al. (115) investigated the putative role the hydroxycarboxylic acid receptor 1 (HCAR1), enriched at the blood-brain barrier (135), as a mediator of lactate-induced central vascularization. They observed that after 7 weeks of wheel-running activity, mice lacking HCAR1 did not show increased VEGFA expression and capillary density in the dentate gyrus or the sensorimotor cortex. Similarly, chronic subcutaneous injections of sodium L-lactate failed to enhance VEGFA expression and vascularization in HCAR1 KO animals, indicating that HCAR1 is essential to mediate the effects of exercise on angiogenesis in specific brain areas. Moreover, in line with the involvement of the phosphatidylinositol 3-kinase/Akt (PI3K/Akt) and extracellular signal-regulated kinase (ERK1/2) signaling pathways in mediating the induction of VEGF (136), both lactate and a selective HCAR1 agonist failed to activate ERK1/2 and Akt in hippocampal slices lacking HCAR1. The authors further defined the location of the HCAR1 protein in the leptomeningeal fibroblast-like cells of the pia mater—the innermost layer of the meninges which supports blood vessel—as well as pericyte-like cells, previously shown to be involved in the control of blood flow and angiogenesis (137).

### Kynurenin and kynurenic acid

The underlying cause of depression is difficult to elucidate as a combination of biological, psychological and environmental factors contribute to the pathogenesis of this disease. Notably however, depressed patients are usually less physically active, and the effectiveness of exercise programs in reducing depressive symptoms has been widely recognized (138, 139). Kynurenine (KYN) is a metabolite of the amino acid L-tryptophan, primarily synthetized in the liver. The degradation of KYN follows two different pathways: one proceeds with the conversion of KYN to 3-hydroxykynurenine (3HK) and quinolinic acid (QUIN), while the alternative pathway converts KYN to kynurenic acid (KYNA). In the brain, the conversion to KYNA is catalyzed by kynurenine aminotransferases (KATs) found in astrocytes. The same enzymes are expressed in different peripheral tissues including skeletal muscle (140, 141). Importantly, evidence suggests that a dysregulation of the KYN pathway is associated with a number of psychiatric disorders such as depression (140, 141). In a study from Agudelo et al. (121), exercise has been shown to activate the expression of KATs through a PGC-1α- and PPARα/δ-dependent pathway in both mouse and human muscles. Consistently, overexpression of PGC-1α and loss of PGC-1α, respectively, resulted in increased and decreased muscle KAT expression levels. The increase in KATs expression in the gain-of-function model shifted KYN metabolism toward enhanced synthesis of KYNA, which is unable to cross the blood-brain barrier, thereby reducing the accumulation of plasma KYN with its neurotoxic effects on the brain. Remarkably, this shift conferred resistance to chronic mild stress-induced depression in mice overexpressing PGC-1α in their muscles. The authors furthermore demonstrated that muscle PGC-1α elevation prevented the alterations in the expression of neurotrophic, synaptic and pro-inflammatory markers in the hippocampus in response to chronic mild stress and, to a lesser extent, exogenous KYN administration. These data suggest that physical exercise could also affect neuroinflammation [reviewed in (142, 143)].

These findings further illustrate how muscle PGC-1α can mediate the benefits of exercise in the crosstalk between skeletal muscle and the brain. Incidentally, a new observation in healthy individuals confirmed that regular endurance training promotes skeletal muscle KATs gene and protein expression, along with enhanced peripheral conversion of KYN to KYNA (122). Moreover, the “detoxifying” potential of skeletal muscle may not be limited to KYNA, as muscle PGC-1α also regulates systemic ketone body levels and can prevent hyperketosis (144).

### β-hydroxybutyrate

The transcriptional regulation of BDNF in the hippocampus at least in part depends on the modulation of histone acetylation (145). By comparing sedentary mice to mice that had running-wheel access for 30 days, Sleiman et al. (116) found that exercise induced changes in class I HDAC expression, a family of proteins that suppress gene expression by deacetylating lysine residues on histone and non-histone proteins. In particular, binding of HDAC2 and HDAC3 to the hippocampal *Bdnf* promoter I and II was reduced in exercising mice, leading to elevated BDNF levels. Interestingly, besides its role as energy source, the ketone body BOHB also acts as an epigenetic signaling molecule by inhibiting class I HDACs (23). Sleiman and colleagues hypothesized that this exercise factor might inhibit the action of the histone deacetylases on the *Bdnf* promoter. They showed that exercise induces BOHB accumulation in the hippocampus and that the direct injection of BOHB into the ventricles of mice led to higher BDNF expression. In addition, application of BOHB to primary neurons decreased HDAC3 binding to the *Bdnf* promoter, whereas the use of a selective HDAC3-inhibitor or the knockdown of HDAC3 by shRNA increased BDNF expression. This effect on BDNF expression appeared specific, as BOHB did not induce *Pgc-1*α, *Fndc5*, or *Err*α mRNA in primary neurons. The authors also found that the increase of BDNF by BOHB led to enhanced glutamate release in hippocampal slices, and that this effect was dependent on TrkB signaling. The potential of BOHB to convey peripheral information to the brain is further illustrated by other findings demonstrating that liver-derived BOHB modulates neural network-based prediction of feeding time in mice (146).

### Muscle *Bmal1*

Sleep is a critical health function that is controlled by complex mechanisms distributed in multiple brain areas such as the cortex, the brain stem and the hypothalamus (147). Circadian clocks are located in nearly every cell of the mammalian body and generate molecular rhythms with a 24-h period, essential to adjust the behavior and physiology of an organism to the external environment (148). In the past, several studies in mice and humans have demonstrated the consequence of genetic clock perturbations on sleep homeostasis (149, 150). In particular, global deletion of the core clock gene *Bmal1* in mice can affect sleep processes, with longer non-rapid eye movement (NREM) sleep time and altered sleep response after a period of sleep loss (151). Recent findings from Ehlen et al. (123) have challenged the common view that sleep is a process that is only influenced by the brain. They found that restoring *Bmal1* expression in the brain of whole-body *Bmal1* KO mice failed to normalize NREM sleep amount to control values, suggesting that *Bmal1* in another tissue might be important to regulate sleep physiology. Indeed, rescuing *Bmal1* in skeletal muscle was sufficient to restore NREM sleep amount as well as sleep recovery after sleep loss. A causative role for muscle *Bmal1* was further supported by the fact that *Bmal1* deletion only in skeletal muscle recapitulated the sleep alterations seen in whole-body *Bmal1* KO mice, whereas overexpression of *Bmal1* in skeletal muscle of wild-type mice renders mice resistant to sleep deprivation.

These results are intriguing for several reasons. First, they suggest for the first time that a single gene in a peripheral tissue can modulate essential centrally-generated sleep processes—whether this depends on the BMAL1 association with CLOCK or other partners in muscle cells has to be investigated. Second, these data imply a muscle-to-brain signaling involved in sleep regulation, either humoral, or, based on the observation that mice lacking *Bmal1* in their muscles exhibit potent metabolic disruptions (152, 153), via metabolites. Third, because restoring *Bmal1* expression in muscle cells does not restore circadian rhythmicity (154), and more importantly here, the diurnal rhythm of NREM sleep, it is possible that the production and secretion of this muscle-derived factor is a time-controlled cellular event. In the same line of thought, it would be interesting to evaluate whether scheduled exercise, in addition to muscle *Bmal1* reintroduction, is able to reinstate sleep phase timing in this mouse model.

## Putative candidates, network and future research

### Cytokines

Contraction-induced release of IL-6 has potent effects on whole-body glucose disposal (105). Interestingly, IL-6 can act locally on muscle cell glucose uptake and fatty acid oxidation in an autocrine/paracrine manner (155). Systemic elevation of IL-6 by exogenous administration or exercise stimulates insulin release from pancreatic cells (156) and mediates fasting-induced free fatty acid mobilization by adipose tissue (157) as an endocrine hormone. Importantly, other cytokines, e.g., IL-8 and IL-15 mRNA, are also upregulated upon exercise in skeletal muscle and have the potential to behave as myokines [reviewed in (112)]. Additional evidence suggests that the murine chemokine CXC ligand 1 (CXCL-1) and the leukemia inhibitory factor (LIF)—belonging to the IL-6 cytokine superfamily—are both produced by contracting muscle cells and may exert local or long-distance actions [reviewed in (158)]. Likewise, *in vitro* data from primary human myotubes further suggest a broader muscle cytokines response to exercise (108). Interestingly, most of these cytokines are neuromodulators when expressed by neurons and glial cells, potentially influencing brain health and disease (159). This is especially evidenced in the context of higher central or peripheral level of pro-inflammatory cytokines, which reduce central BDNF expression and thus impact on neurogenesis and neurotransmitter release (160, 161). It is currently unclear whether all of these cytokines are released from muscle fibers or from resident immune cells in this tissue. Moreover, a potential crosstalk between muscle and brain remains to be shown: potentially, cytokine signaling at the blood-brain interface might be important to modulate aspects of brain physiology such as sleep, memory or feeding behavior.

### FGF21

Fibroblast growth factor 21 (FGF21) is an endocrine hormone expressed by several tissues including skeletal muscle, adipose tissue and pancreas. The main source of FGF21 is the liver during prolonged fasting, presumably via PPARα- and PGC-1α-dependent mechanisms, resulting in a modulation of systemic energy balance, insulin sensitivity, hepatic gluconeogenesis and glucocorticoid levels (162). Importantly, FGF21 can cross the blood-brain barrier and is found in both human and mouse cerebrospinal fluid (163, 164). Moreover, FGF21 signals in the brain through its co-receptor, βKlotho, as well as the FGF receptor-1, located in the hypothalamus (117, 162). Proposed effects for FGF21 in the nervous system are the modulation of the sympathetic nerve input to brown adipose tissue, the control of circadian behavior, and neuroprotection (117, 119, 165). Studies also suggest that FGF21 can be produced by muscle cells in certain context involving e.g., the activation of AKT or the mTORC1 pathway (166–169). Pereira et al. (170) demonstrated that a muscle deficiency of the mitochondrial fusion protein optic atrophy 1 (OPA1) alters mitochondrial dynamics and increases endoplasmic reticulum (ER) stress, thereby promoting muscle-FGF21 release and rendering mice resistant to age- and diet-induced obesity and insulin resistance. The molecular mechanism by which ER-stress activates FGF21 is not clear, but could involve the transcriptional co-regulator PGC-1α (171, 172). Of interest, one study showed increase serum FGF21 levels in mice and humans after exercise, yet likely of liver and not skeletal muscle or adipose tissue origin (118). In summary, FGF21 can be considered as an hepatokine, adipokine, and potentially a myokine, and has various effects at the whole-body level including the nervous system. Thereby, future research should investigate the putative involvement of FGF21 in the muscle-brain crosstalk, especially during exercise.

### Inter-cell crosstalk

Skeletal muscle is a part of a larger system which also encompasses bones, tendons, ligaments, cartilage, joints, nervous and connective tissues. In particular, beyond their mechanical coupling, bone and muscle cells can communicate at the biochemical and molecular levels underlining a potential paracrine/endocrine crosstalk. Different studies demonstrated an effect of muscle-specific gene deletion or overexpression on bone physiology [reviewed in (173, 174)], and a close correlation between bone and muscle deterioration during aging (175). As in other cell types, different bone cell types have the capacity to release factors into the circulation (176). For instance, the osteoblast-secreted protein osteocalcin is essential to regulate energy metabolism by increasing insulin sensitivity via the stimulation of adiponectin release by adipocytes (177). Intriguingly, mouse and human data showed that circulating osteocalcin levels are increased upon exercise, and greatly diminished during aging. Moreover, bone-derived osteocalcin signals to muscle cells and favors the uptake and utilization of glucose and fatty acids in addition to stimulate muscle secretion of IL-6, which again feedbacks to bone cells to enhance osteocalcin production (178). Mosialou et al. (179) revealed that the bone-derived glycoprotein lipocalin 2 (LCN2), a previously identified component of the immune response (180), crosses the blood-brain barrier and binds to the melanocortin 4 receptor (MC4R) in the paraventricular and ventromedial neurons of the hypothalamus to inhibit food intake. Lipocalin 2 is also produced in muscle cells under the control of PGC-1α (181).

These findings implicate crosstalk of skeletal muscle with more dispersed peripheral cells. For instance, physical exercise induces the secretion of the adipose-derived hormone adiponectin, which enhances hippocampal neurogenesis and has potent antidepressant effects (182). Interestingly, a role for muscle IL-15 has been suggested in the regulation of adiponectin production (183).

### The circadian muscle secretome

Circadian clocks control myriad of processes such as gene regulation, protein synthesis and export, enzyme activity, cell signaling, nutrient accumulation, on a 24-h time scale. Moreover, clock proteins are cellular sensors, in that they can integrate specific changes in the external or internal environment (184). Chronic circadian disruption by irregular sleep-wake cycles or meal timing has been associated with obesity and other metabolic conditions, cancer and inflammation, sleep as well as cognitive and mental disorders (185–188). In skeletal muscle, the molecular clock has been linked to cell growth and repair, autophagy, insulin sensitivity, lipid homeostasis, mitochondrial metabolism and respiration (152, 189–191). Moreover, scheduled exercise is a potent stimulus to regulate circadian timing in skeletal muscle (192, 193). Interestingly, human primary myotubes harbor a cell-autonomous circadian clock involved in the basal release of cytokines (e.g., IL-6, IL-8) and other proteins (e.g., VEGF, FABP) as identified by ELISA and multiplex assays (194). Intriguingly, *Pgc-1*α transcript oscillates in skeletal muscle and PGC-1α can co-activate RORα to induce the expression of *Bmal1* in primary hepatocytes (195). Furthermore, genetic ablation of *Bmal1* from muscle cells gave evidence that a peripheral clock function is essential for centrally-controlled sleep processes (123). Thereby, it is likely that a transcriptional regulatory network, involving circadian nuclear receptors and the coactivator protein PGC-1α coordinates the time-of-day-dependent accumulation of myokines in muscle cells and secretion into the circulation. Future work should for instance evaluate the temporal phase relationship between core clock components and muscle-derived factors. Moreover, if part of the muscle secretome is gated by the circadian clock as in other endocrine organs (196), there are certainly time windows during which exercise can exert maximal effects on brain physiology.

Altogether, there is a likely bidirectional influence between circadian and exercise pathways in muscle cells—whether this is important for skeletal muscle endocrine function requires further investigation. In any case, at the organismal level, circadian clocks clearly provide a molecular basis by which endocrine signaling—i.e., from the production and secretion of an endocrine factor to the activation of a signaling pathway—can be achieved in a timely fashion. Given the conserved relation between circadian clock and neuronal function (197–201), time-based physical activity interventions may potentiate the health benefits of exercise.

## Conclusion

**Figure 1:**
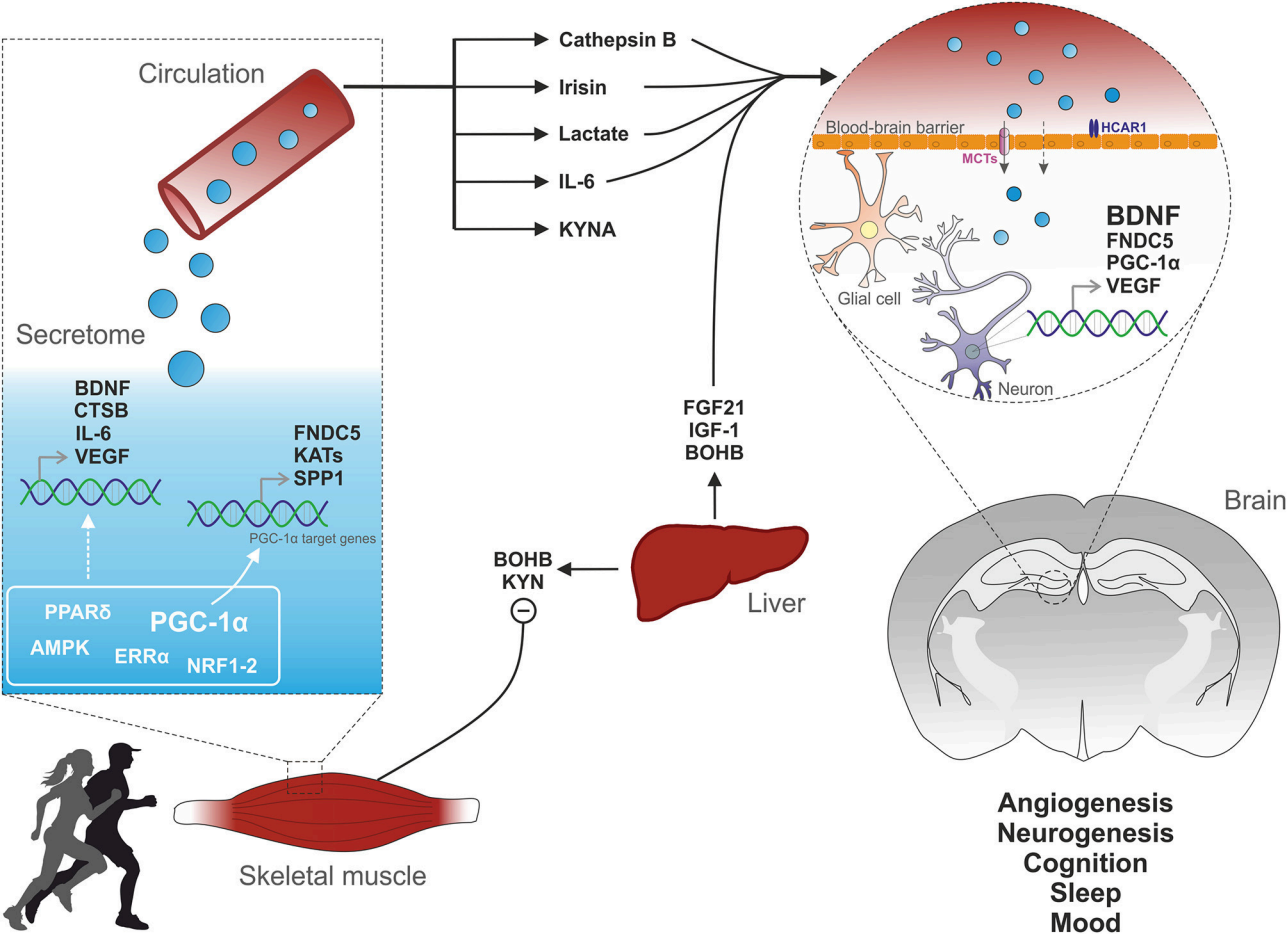
Muscle-brain crosstalk. Physical exercise activates specific cellular pathways in muscle cells. For instance, PGC-1α activation induces the expression of FNDC5, which is cleaved to irisin and released into the circulation. PGC-1α elevation also leads to the biosynthesis of kynurenine aminotransferases (KATs) which converts liver-derived KYN to KYNA, thus preventing its toxic accumulation into the brain. The endocrine property of muscle cells is further illustrated by the release of cytokines (e.g., IL-6) or metabolites (e.g., lactate). Physical activity also promotes the production and release into the blood of various factors from non-muscle tissues such as the liver. Subsequently, muscle- and liver-derived molecules enter the brain and signal on receptors located on endothelial, glial or neuronal cells, thereby triggering the expression of VEGF and BDNF, key regulators of cerebral vascularization and plasticity.

Genetic engineering and the relentless motivation of captive rodents to be active on an exercise wheel have given scientists the opportunity to identify several muscle-derived signaling factors (Figure 1). At the central level, BDNF is a crucial mediator of the effects of exercise for the modulation of synaptic transmission, neurogenesis and memory function. In skeletal muscle, PGC-1α exerts a strong “detoxifying” potential and is an effective regulator of the muscle secretome, essential for exercise adaptations and the regulation of central function such as memory and mood. However, the timing and regulation of myokine production and secretion are still poorly understood. For many putative myokines, a thorough characterization and validation is still lacking [discussed in (14)]. Moreover, discrepancies in the identification of putative myokines have been emerging in studies in cell-based compared to *in vivo* models (202). Finally, for most of these factors it is unclear whether peripheral or central production is mediating the beneficial effects on the brain. Nevertheless, the strong epidemiological evidence of exercise as a powerful intervention to prevent and treat various pathologies warrants a careful dissection of the signals, networks and mediators of the inter-organ crosstalk originating from skeletal muscle. This is of particular importance for neurological disorders, where therapeutic options are scarce and desperately needed.

## Author contributions

All authors listed have made a substantial, direct and intellectual contribution to the work, and approved it for publication.

### Conflict of interest statement

The authors declare that the research was conducted in the absence of any commercial or financial relationships that could be construed as a potential conflict of interest.

## Abbreviations

3HK: Hydroxykynurenine
AICAR: 5-Aminoimidazole-4-carboxamide ribonucleotide
AMPK: AMP-dependent kinase
BAIBA: β-Aminoisobutyric acid
BDNF: Brain-derived neurotrophic factor
BMAL1: Also known as Aryl hydrocarbon receptor nuclear translocator-like protein 1 (Arntl)
BOHB: β-Hydroxybutyrate
CREB: Cyclic-AMP-responsive-element-binding protein
CTSB: Cathepsin B
CXCL-1: CXC ligand 1
DCX: Doublecortin
ER: Endoplasmic reticulum
ERK1/2: Extracellular signal-regulated kinase
ERRα: Estrogen-related receptor α
FABP: Fatty-acid-binding protein
FGF21: Fibroblast growth factor 21
FNDC5: Fibronectin type III domain-containing protein 5
FXR: Farnesoid X receptor
GLUT4: Glucose transporter 4
HCAR: Hydroxycarboxylic acid receptors
HDAC: Histone deacetylase
IGF-1: Insulin-like growth factor 1
IL: Interleukin
KAT: kynurenine aminotransferase
KYN: Kynurenine
KYNA: Kynurenic acid
LCN2: Lipocalin 2
LIF: Leukemia inhibitory factor
LXRs: Liver X receptor
MC4R: Melanocortin 4 receptor
MCT: Monocarboxylate transporter
mTOR: Mechanistic target of rapamycin
NO: Nitric oxide
NREM: Non-rapid eye movement
NRF: Nuclear respiratory factor
OPA1: mitochondrial fusion protein optic atrophy 1
PGC-1α: Peroxisome-proliferator activated receptor γ coactivator 1α
PI3K/Akt: Phosphatidylinositol 3-kinase/Akt
PKA: Protein kinase A
PPAR: Peroxisome-proliferator activated receptor
QUIN: Quinolinic acid
REV-ERBα: also known as nuclear receptor subfamily 1 group D member 1 (Nr1d1)
RORα: RAR-related orphan receptor alpha
ROS: Reactive oxygen species
RXR: Retinoid X receptor
SIRT1: Silent mating type information regulation 2 homolog
SPP1: Secreted phosphoprotein 1
TrkB: Tropomyosin receptor kinase B
VEGF: Vascular endothelial growth factor.
